# Primary splenic abscess in an adult female patient: a case report

**DOI:** 10.1186/s40792-024-01849-2

**Published:** 2024-03-01

**Authors:** Burce Isik, Matthew G. Davey, Sarah Gaffney, Patrick J. Stapleton, Javier Mohigefer, Efthymios Koutroumanos

**Affiliations:** 1 University of Limerick School of Medicine, Co. Limerick, Republic of Ireland; 2 Department of Surgery, University Hospital Limerick, Dooradoyle, Co. Limerick, Republic of Ireland; 3 Department of Anaesthesia and Intensive Care, University Hospital Limerick, Dooradoyle, Limerick, Ireland; 4 Department of Microbiology, University Hospital Limerick, Dooradoyle, Co. Limerick, Republic of Ireland; 5 Department of Histopathology, University Hospital Limerick, Dooradoyle, Co. Limerick, Republic of Ireland

**Keywords:** Primary splenic abscess, Splenic infarct, Laparotomy, Splenectomy, Acute abdomen

## Abstract

**Background:**

Primary splenic abscess is rare and typically presents in patients who are immunocompromised. We present a case of a 47-year-old apparently immunocompetent female patient who was diagnosed with primary splenic abscess from a *Salmonella* Typhimurium infection following emergency laparotomy.

**Case presentation:**

A 47-year-old female patient presented with subjective fever and severe epigastric and left flank pain. She was treated empirically with intravenous piperacillin/tazobactam and gentamicin and was resuscitated with intravenous crystalloid infusion. A radiological diagnosis of splenic infarct secondary to splenic artery aneurysm superimposed with splenic abscess was presumed, however at emergency laparotomy, primary splenic abscess was identified. This abscess had eroded the left hemidiaphragm and had ruptured the splenic capsule leading to intra-abdominal pus in the pelvis which on culture grew *Salmonella* Typhimurium. A splenectomy and primary repair of the left hemidiaphragm were performed, with postoperative pancreatitis diagnosed following the procedure. After intensive care treatment, this patient made a full recovery.

**Conclusion:**

This case of primary splenic abscess was treated successfully with a combination of surgery (i.e.: splenectomy and surgical drainage), prolonged antimicrobial therapy, and intensive care in the perioperative period.

## Introduction

Primary splenic abscess is a rare pathology, with an incidence of 0.14–0.70% reported in the literature [1]. The robust immune function of a healthy spleen protects the parenchyma from colonization by microorganisms, rendering abscess formation uncommon unless in the setting of immunosuppression. Arriving at the diagnosis of primary splenic abscess can be challenging, due to non-specific signs and symptoms, which mimic other syndromes, including pyelonephritis and pneumonia. Herein, we present an unusual case of a female patient with no known immunodeficiency presenting with severe abdominal pain due to primary splenic abscess. We appreciate that this is not the first case that has ever demonstrated primary splenic abscess. However, it is certainly unique in its presentation since it has not been picked up for 2 weeks by primary and tertiary care providers.

## Case report

A 47-year-old female patient presented to the Emergency Department (ED) with a 14-day history of severe epigastric and left flank pain radiating to her groin. Seven days prior she was treated by her general practitioner with empiric amoxicillin/clavulanic acid for suspected pyelonephritis. At home, the patient reported ongoing subjective fevers, increasing abdominal pain, nausea and vomiting. This patient’s past medical and surgical history was significant for benign idiopathic intracranial hypertension for which a lumbar peritoneal (LP) shunt was inserted 17 years previously, four cerebral aneurysms requiring coiling by interventional radiology with the past 10 years, a congential strabismus (Duane’s syndrome) and a 15-pack-year history of smoking. There was no history of recurrent or severe infections. There was a significant family history of vascular disease with the patient’s mother experiencing recurrent strokes commencing in her late 30s and brother an aortic aneurysm in his 50s.

On arrival to the ED, the patient was diaphoretic and mottled in her distant limbs. Her temperature was 39 degrees Celsius and her oxygen saturation was 94% on room air, heart rate was 117 beats per minute and she was tachypneic with a respiratory rate of 23. Physical examination revealed a soft but tender abdomen, worse in her left flank and epigastric area, without evidence of peritonism, guarding or rigidity.

**Fig. 1:**
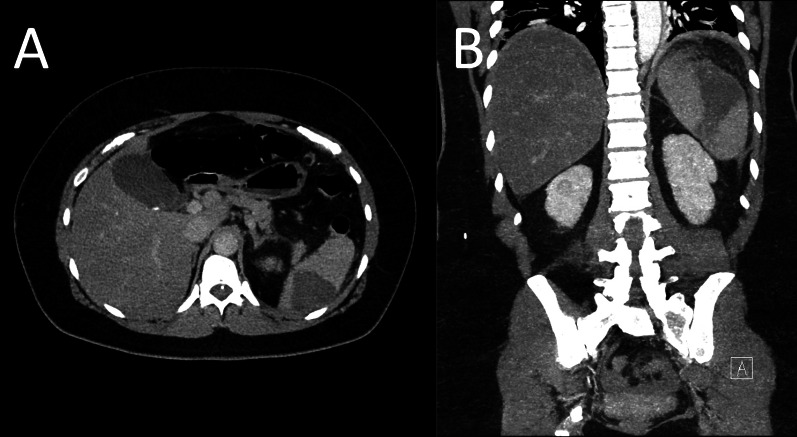
**A** Axial and **B** coronal commuted tomography views demonstrating the abnormality in the spleen

Laboratory findings were significant for a venous lactate of 2.0 mmol/L, white blood cell count of 22.1 × 10^9^/L, neutrophil count of 16.7 × 10^9^/L, C-reactive protein level of 389 mg/L and d-dimer > 4 μg/mL. A urine dipstick demonstrated leukocytes without bacteria. This patient was resuscitated using supplementary oxygenation and intravenous (IV) crystalloid infusion before being commenced on broad spectrum IV antimicrobials (piperacillin/tazobactam 4.5 g every 8 h and gentamicin 320 mg stat dose). A urinary catheter was inserted, before the patient was made nil by mouth and an urgent surgical consultation was sought. Thereafter, dual phase computed tomography of the abdomen and pelvis was performed, which demonstrated large volume hypoattenuation involving the mid-to-upper pole of the spleen suggestive of subacute infarct, with rim enhancement and marked peri-splenic stranding, with associated localized peritonitis indicating superimposed abscess formation (Fig. 1A and B). A thrombosed 14 mm splenic artery aneurysm was noted and perceived to be the likely aetiology of the presumed splenic infarct. A small volume of free fluid was also noted in the pelvis with hazy attenuation in the omentum and mesenteric fat suggestive of generalized peritonitis.

The radiological diagnosis was splenic infarct secondary to splenic artery aneurysm with superimposed splenic abscess presumed. The local Haematology and Vascular surgery service were consulted and the patient was commenced on an unfractionated heparin infusion, which was stopped 4 h prior to the patient proceeding to theatre. Blood cultures collected on admission flagged positive for growth of Gram-negative bacilli in both bottles after 13 h incubation; antibiotic treatment was escalated to meropenem pending identification and susceptibility test results.

**Fig. 2:**
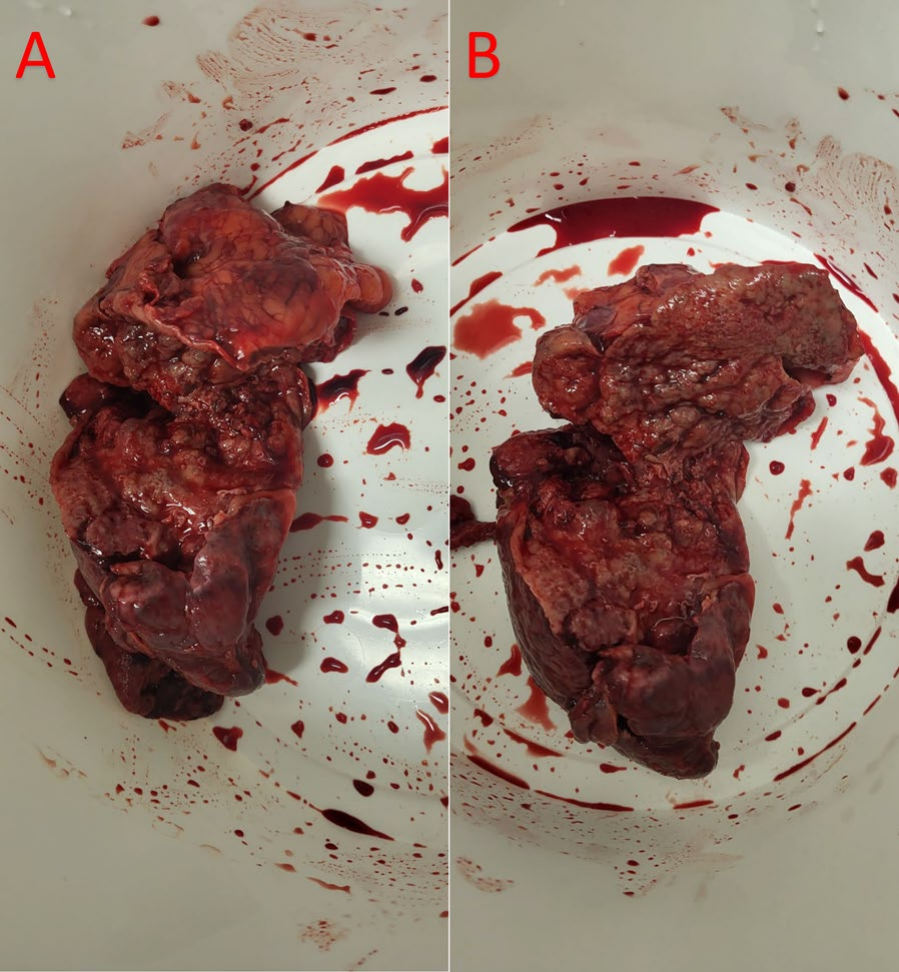
Postoperative clinical photography of resected primary splenic abscess, with abscess cavity **A** closed and **B** open

At surgery, this patient initially underwent a diagnostic laparoscopy with a Veress needle used to establish the pneumoperitoneum, before two 11-mm trocars and one 5-mm trocar were placed. An early decision was made for conversion to midline laparotomy, due to excessive free purulent fluid and dense adhesions limiting laparoscopy of the greater sac. Pus was suctioned from the pelvis and samples sent for culture. Adhesions were noted transversing from the greater omentum to the spleen. It was noted that the spleen was completely perfused, however there was an obvious primary abscess within the superior and middle poles of the spleen. Moreover, the superior pole of the abscess was firmly adherent to the inferior left hemidiaphragm, with fistulation into the left pleural cavity. A splenectomy was performed (Fig. 2A and B), with primary repair of the diaphragm using 0-prolene suture performed thereafter, leading to significant improvement in patient ventilation. This repair was then tested by filling the peritoneum with normal saline wash, combined with Valsalva manoeuvre, which was negative for any obvious fistulation or ‘air leak’. Care was taken throughout to preserve the LP shunt. Two Robinson drains were inserted into abdominal cavity, one in the pelvis and another transversing the splenic bed. The abdominal fascia was then closed with 0-loop polydioxanone sutures, with staples applied to skin.

Postoperatively, the patient was transferred back to the intensive care unit (ICU) intubated and ventilated. She remained on 20 mcg/min of noradrenaline which was slowly weaned over the subsequent 24 h. After 24 h she was successfully extubated on to high-flow nasal oxygen at 40L/40% and the remainder of her vasopressor support was weaned off. Thereafter, she was escalated to IV caspofungin 70 mg once daily (OD), Gentamicin 280 mg OD, continuous Vancomycin infusion at 6 mL/h and continuation of meropenem 2 g three times daily. Drain output was monitored closely in the postoperative setting and an expected pancreatitis was diagnosed due to a splenic drain serum amylase level elevated at 3700 U/L.

Blood cultures from admission and the intra-operative peritoneal fluid yielded *Salmonella Typhimurium* that was resistant to amoxicillin and co-amoxiclav and susceptible to all other agents tested. Taking the positive cultures and her LP shunt into account, her antimicrobials were rationalized to Ceftriaxone 2 g twice daily intravenously. Phenoxymethylpenicillin 666 mg OD orally was added for post-splenectomy antibiotic prophylaxis and immunization with Pneumococcal, Meningococcal, *Haemophilus influenza* type b, Influenza and SARS-CoV-2 vaccines was administered at intervals following surgery.

Thereafter, this patient continued to improve clinically. She had a left basal pleural effusion (attributed to the primary diaphragmatic repair) and a postoperative pancreatitis, both of which resolved within 14 days of surgery. HIV testing was negative. The patient was referred to the Infectious Diseases team after discharge from ICU for consideration of extended duration of IV antibiotics (up to 6 weeks) as outpatient in view of the LP shunt and aneurysmal coils and for consideration of genetic testing for possible occult immunodeficiency syndromes such as STAT-1 gain of function mutation.

**Fig. 3:**
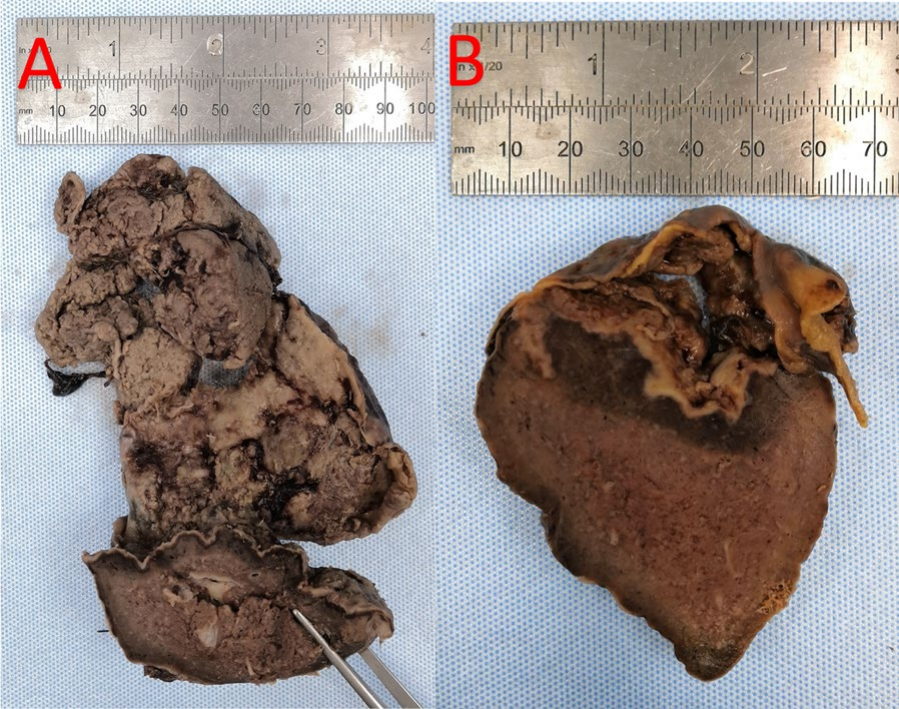
Histopathological specimen demonstrating final macroscopic splenectomy specimen demonstrated a central ruptured abscess with abundant fibrinonecrotic material (**A**), with almost all the spleen covered in fibrin (**B**)

The final macroscopic splenectomy specimen demonstrated a central ruptured abscess with abundant fibrinonecrotic material (Fig. 3A), with almost all the spleen covered in fibrin (Fig. 3B).

**Fig. 4:**
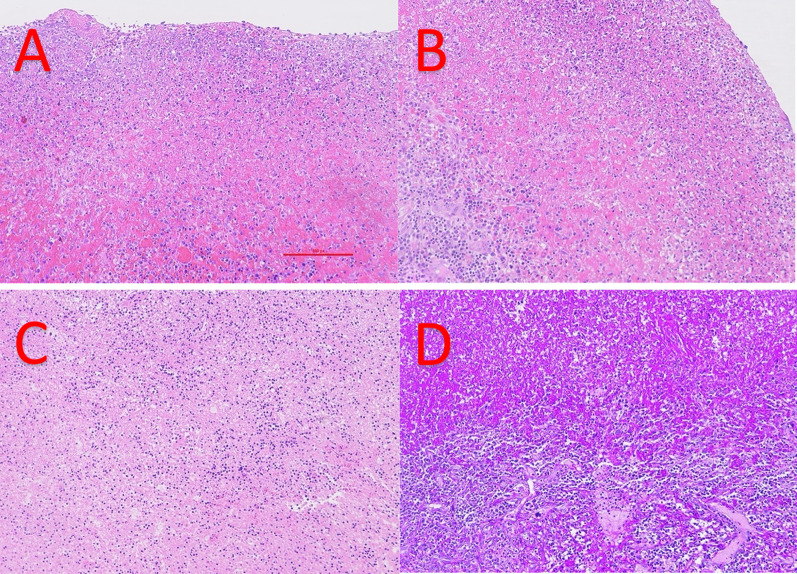
Microscopically, a marked acute inflammatory infiltrate with abundant fibrinoleukocytic superficial material was identified on the splenic surface (**A** and **B**), with abundant abscess formation (**C**), with no periodic acid schiff (PAS)-positive organisms detected (**D**)

Microscopically, the local Department of Histopathology noted that there was a marked acute inflammatory infiltrate present in the spleen, with abundant fibrinoleukocytic superficial material identified on the splenic surface (Fig. 4A and B). It was also noted that there was abundant abscess formation (Fig. 4C), with no periodic acid Schiff (PAS)-positive organisms detected (Fig. 4D).

## Discussion

Herein, we describe the case of primary splenic abscess in an apparently immunocompetent female adult patient who was treated empirically for a priori diagnosis of pyelonephritis, which was presumed given her pyrexia, history of left flank pain with associated nausea, and an elevated biochemical profile. This case reminds the clinician to be suspicious of this clinical presentation, particularly when the patient’s symptoms are seemingly resistant to a prolonged course of antimicrobial therapy, in the absence any obvious stigmata supportive of other intra-abdominal pathology.

The diagnosis of primary splenic abscess is often delayed, likely due to its tendency to ‘mimic’ other pathologies, as occurred in the current case. Nevertheless, it is also important to note that there are several previous case reports of splenic abscesses, which typically tend to occur secondary to locoregional infections, such as in cases of infective endocarditis, hematological disorders, and in the setting of severe acquired immunodeficiency syndrome [2]. Thorough immune investigations were performed in our case but yielded no results indicative of immunosuppressive. The importance of the current case is to highlight the complexities involved in diagnosing primary splenic abscess, particularly due to the presence of other ‘red herrings’ in the clinical presentation of this patient; for example, the unexplained respiratory compromise (which was later attributed to the diaphragmatic involvement of the abscess), the radiological diagnosis of splenic infarct (assumed to be secondary to a 14 mm splenic artery aneurysm), the LP shunt in situ (considered as a potential cause for abdominal sepsis, as described by Llenas-García et al. where a 41-year-old patient with a LP catheter in situ developed a secondary splenic abscess from the shunt which grew *Propionibacterium* species [3]), and also a urinalysis performed in the ED which demonstrated leukocytes. Ultimately, the formal diagnosis could not be clinically, radiologically or biochemically arrived at until primary splenic abscess was identified during laparotomy.

Importantly, this patient was treated successfully with a combination of surgery (i.e.: splenectomy and surgical drainage), antimicrobial therapy, and ICU care in the perioperative period, in accordance with ‘gold standard’ recommendations [4]. While previous authors have reported management of splenic abscess resolving with percutaneous drainage only [3], this case was complicated given the uncertainty regarding splenic infarction, abscess rupture (leading to intra-abdominal sepsis), and the local erosion of left hemidiaphragm, rendering definitive surgical management the safest means for managing this patients care.

Splenic abscesses due to *Salmonella* species are reported to occur in up to 2% of patients with typhoid fever [5]. The route of *Salmonella* Typhimurium entry is uncertain in this case. However, the team providing medical care to the patient believes that it is probably through the gastrointestinal tract and it was subclinical until it manifested in the spleen. It is important to note that although pyelonephritis was a provisional diagnosis at the time, there was no evidence of *Salmonella* Typhimurium in the urine despite rigorous culture and sensitivity. Therefore, it is unlikely that this was secondary to pyelonephritis. Our case highlights an unusual presentation of *Salmonella* Typhimurium infection, as well as one of its rare complications.

## Conclusion

In conclusion, we present the case of an apparently immunocompetent female presenting with a splenic abscess secondary to *Salmonella* Typhimurium bacteremia. We highlight the challenges in diagnosis as well as the complexities and multidisciplinary approach to its management.

## Data Availability

Not applicable.

## Abbreviations

ED: Emergency department
LP: Lumbar peritoneal
IV: Intravenous
ICU: Intensive care unit
OD: Once daily
PAS: Periodic acid Schiff
